# A General Formula for Fan-Beam Lambda Tomography

**DOI:** 10.1155/IJBI/2006/10427

**Published:** 2006-02-02

**Authors:** Hengyong Yu, Ge Wang

**Affiliations:** CT/Micro-CT Laboratory, Department of Radiology, The University of Iowa, Iowa City, IA 52242, USA

## Abstract

Lambda tomography (LT) is to reconstruct a gradient-like image of an object only from local projection data. It is potentially an important technology for medical X-ray computed tomography (CT) at a reduced radiation dose. In this paper, we prove the first general formula for exact and efficient fan-beam LT from data collected along any smooth curve based on even and odd data extensions. As a result, an LT image can be reconstructed without involving any data extension. This implies that structures outside a scanning trajectory do not affect the exact reconstruction of points inside the trajectory even if the data may be measured through the outside features. The algorithm is simulated in a collinear coordinate system. The results support our theoretical analysis.


Hindawi Publishing Corporation
International Journal of Biomedical Imaging
Volume 2006, Article ID 10427, Pages 1–9
DOI 10.1155/IJBI/2006/10427

### A General Formula for Fan-Beam Lambda Tomography

**Hengyong Yu and Ge Wang**

*CT/Micro-CT Laboratory, Department of Radiology, The University of Iowa, Iowa City, IA 52242, USA*

Received 4 September 2005; Revised 12 November 2005; Accepted 12 November 2005

Recommended for Publication by Guowei Wei

Lambda tomography (LT) is to reconstruct a gradient-like image of an object only from local projection data. It is potentially an important technology for medical X-ray computed tomography (CT) at a reduced radiation dose. In this paper, we prove the first general formula for exact and efficient fan-beam LT from data collected along any smooth curve based on even and odd data extensions. As a result, an LT image can be reconstructed without involving any data extension. This implies that structures outside a scanning trajectory do not affect the exact reconstruction of points inside the trajectory even if the data may be measured through the outside features. The algorithm is simulated in a collinear coordinate system. The results support our theoretical analysis.

Copyright © 2006 H. Yu and G. Wang. This is an open access article distributed under the Creative Commons Attribution License, which permits unrestricted use, distribution, and reproduction in any medium, provided the original work is properly cited.

### 1. INTRODUCTION

Since the ionizing radiation may induce cancers and genetic damages in the patient, it is highly desirable to minimize the X-ray dose during a CT scan. For that purpose, region-of-interest- (ROI-) based tomography has been extensively studied, which reconstructs a local image from truncated projection data but suffers from image cupping and intensity shifting artifacts since such a CT problem does not have a unique solution [1]. Lambda tomography (LT) was proposed as a novel alternative [2–9]. Let $\mathbf{x}$ and $\boldsymbol{\xi}$ represent two-dimensional (2D) vectors, $f(\mathbf{x})$ a 2D bounded function with a compact support, and $\hat{f}(\boldsymbol{\xi})$ the corresponding Fourier transform, we have

$$\hat{f}(\boldsymbol{\xi}) = \int_{\mathbb{R}^2} f(\mathbf{x}) e^{-i\mathbf{x}\cdot\boldsymbol{\xi}} d\mathbf{x},$$
$$f(\mathbf{x}) = \frac{1}{(2\pi)^2} \int_{\mathbb{R}^2} \hat{f}(\boldsymbol{\xi}) e^{i\mathbf{x}\cdot\boldsymbol{\xi}} d\boldsymbol{\xi}, \quad (1)$$

where $\mathbb{R}^2$ denotes the 2D space. Let $\Lambda$ be the so-called Calderon operator defined as

$$\widehat{\Lambda f}(\boldsymbol{\xi}) = \|\boldsymbol{\xi}\| \hat{f}(\boldsymbol{\xi}). \quad (2)$$

LT is to reconstruct such a gradient-like function $\Lambda f(\mathbf{x})$ only from directly involved projection data.

Traditionally, LT is performed in the framework of the Radon transform [8]. In that context, an LT image can be easily reconstructed from Radon data. However, in practical applications, we typically obtain fan-beam or cone-beam projections with a source moving along a scanning curve. In 1993, Louis and Maass proposed an algorithm [7] to reconstruct an LT image approximately from cone-beam data. The main idea is to perform a 3D Laplace transform on weighted backprojection data [6, 7]. The accuracy of their algorithm depends on the scanning curve. If the trajectory is a circle, it was proved that the reconstructed image becomes exact when the scanning radius approaches infinity. Also, Anastasio et al. developed an approximate local fan-beam FBP algorithm for megavoltage imaging [2]. However, up till now, there are no exact and efficient general algorithms for fan-beam or cone-beam LT. On the other hand, recently there are some remarkable results on exact CT reconstruction from data acquired along any smooth scanning curve [10–20]. Therefore, we are motivated to design theoretically exact, computationally efficient, and practically flexible LT algorithms to reconstruct $\Lambda f(\mathbf{x})$ from fan-beam or cone-beam data.

In this paper, we derive the first general formula for exact and efficient fan-beam LT from data collected along any smooth curve. In Section 2, we present our main result and prove it based on even and odd data extensions. In Section 3, we describe the implementation details for collinear detector geometry. In Section 4, we present numerical simulation results. In Section 5, we discuss relevant issues and make a conclusion.

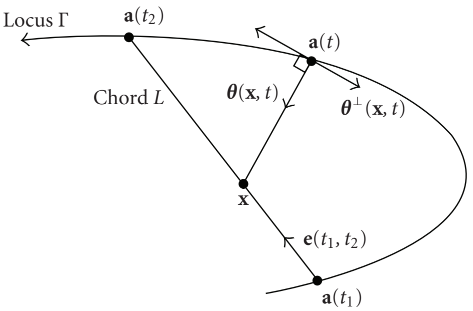

Figure 1: Global coordinate system and variables for fan-beam lambda tomography.

### 2. LT FORMULA AND ITS PROOF

### 2.1. Main result

Let $\mathbb{S}$ represent the unit circle in $\mathbb{R}^2$. Assume that $\Gamma \subset \mathbb{R}^2$ is a differentiable curve parameterized by $\mathbf{a}(t)$, $t \in \mathbb{R}$, and $f$ a bounded function with a compact support $\Omega \subset \mathbb{R}^2 \setminus \Gamma$. A fan-beam projection of $f$ along a scanning trajectory $\Gamma$ is

$$D_f(\mathbf{a}, \boldsymbol{\theta}) = \int_0^\infty ds f(\mathbf{a} + s\boldsymbol{\theta}), \quad (\mathbf{a}, \boldsymbol{\theta}) \in \Gamma \times \mathbb{S}. \tag{3}$$

As shown in Figure 1, a chord $L$ is defined as a line segment with two endpoints $\mathbf{a}(t_1)$ and $\mathbf{a}(t_2)$ on $\Gamma$, and the unit vector along $L$ is

$$\mathbf{e}(t_1, t_2) = \frac{\mathbf{a}(t_2) - \mathbf{a}(t_1)}{||\mathbf{a}(t_2) - \mathbf{a}(t_1)||}. \tag{4}$$

For any point $\mathbf{x} \in L$ and $\mathbf{a}(t) \in \Gamma$, let us introduce the unit vector

$$\boldsymbol{\theta}(\mathbf{x}, t) = \frac{\mathbf{x} - \mathbf{a}(t)}{||\mathbf{x} - \mathbf{a}(t)||}. \tag{5}$$

Let $(\cdot)$ represent the inner product, and let $\boldsymbol{\theta}^\perp(\mathbf{x}, t)$ be a vector perpendicular to $\boldsymbol{\theta}(\mathbf{x}, t)$. Clearly, $\boldsymbol{\theta}^\perp(\mathbf{x}, t)$ is uniquely determined by $\boldsymbol{\theta}(\mathbf{x}, t)$ in the 2D space up to a directional flip. Our main contribution is summarized in the following theorem.

**Theorem 1.** *Let $L$ be a chord from $\mathbf{a}(t_1)$ to $\mathbf{a}(t_2)$ along a differentiable general curve $\Gamma$, $\mathbf{x} \in L$, and $\mathbf{x} \notin \Gamma$. Considering a smooth function $f(\mathbf{x})$ with a compact support,*

$$\Lambda f(\mathbf{x}) = -\frac{1}{2\pi} PV \int_{t_1}^{t_2} dt \frac{\operatorname{sgn}(\mathbf{e} \cdot \boldsymbol{\theta}^\perp)}{||\mathbf{x} - \mathbf{a}(t)|| \cdot (\mathbf{a}'(t) \cdot \boldsymbol{\theta}^\perp)} \times \left( \frac{\partial^2}{\partial q^2} (D_f(\mathbf{a}(q), \boldsymbol{\theta})) \Big|_{q=t} - \frac{(\mathbf{a}''(t) \cdot \boldsymbol{\theta}^\perp)}{(\mathbf{a}'(t) \cdot \boldsymbol{\theta}^\perp)} \frac{\partial}{\partial q} (D_f(\mathbf{a}(q), \boldsymbol{\theta})) \Big|_{q=t} \right), \tag{6}$$

*where $\mathbf{e} = \mathbf{e}(t_1, t_2)$, $\boldsymbol{\theta} = \boldsymbol{\theta}(\mathbf{x}, t)$, $\boldsymbol{\theta}^\perp = \boldsymbol{\theta}^\perp(\mathbf{x}, t)$, $\mathbf{a}'(t) = d\mathbf{a}(t)/dt$, $\mathbf{a}''(t) = d^2\mathbf{a}(t)/dt^2$, and "PV" represents the principle value integral.*

To prove Theorem 1, let us define the even and odd extensions of fan-beam data as

$$D_f^\pm(\mathbf{a}, \boldsymbol{\theta}) = D_f(\mathbf{a}, \boldsymbol{\theta}) \pm D_f(\mathbf{a}, -\boldsymbol{\theta}). \tag{7}$$

Since $D_f(\mathbf{a}, \boldsymbol{\theta}) = (1/2)(D_f^+(\mathbf{a}, \boldsymbol{\theta}) + D_f^-(\mathbf{a}, \boldsymbol{\theta}))$, we can prove Theorem 1 by showing that

$$\Lambda f(\mathbf{x}) = -\frac{1}{2\pi} PV \int_{t_1}^{t_2} dt \frac{\operatorname{sgn}(\mathbf{e} \cdot \boldsymbol{\theta}^\perp)}{||\mathbf{x} - \mathbf{a}(t)|| \cdot (\mathbf{a}'(t) \cdot \boldsymbol{\theta}^\perp)} \times \left( \frac{\partial^2}{\partial q^2} (D_f^+(\mathbf{a}(q), \boldsymbol{\theta})) \Big|_{q=t} - \frac{(\mathbf{a}''(t) \cdot \boldsymbol{\theta}^\perp)}{(\mathbf{a}'(t) \cdot \boldsymbol{\theta}^\perp)} \frac{\partial}{\partial q} \left(D_f^+(\mathbf{a}(q), \boldsymbol{\theta})\right) \Big|_{q=t} \right), \tag{8}$$

$$\Lambda f(\mathbf{x}) = -\frac{1}{2\pi} PV \int_{t_1}^{t_2} dt \frac{\operatorname{sgn}(\mathbf{e} \cdot \boldsymbol{\theta}^\perp)}{||\mathbf{x} - \mathbf{a}(t)|| \cdot (\mathbf{a}'(t) \cdot \boldsymbol{\theta}^\perp)} \times \left( \frac{\partial^2}{\partial q^2} (D_f^-(\mathbf{a}(q), \boldsymbol{\theta})) \Big|_{q=t} - \frac{(\mathbf{a}''(t) \cdot \boldsymbol{\theta}^\perp)}{(\mathbf{a}'(t) \cdot \boldsymbol{\theta}^\perp)} \frac{\partial}{\partial q} (D_f^-(\mathbf{a}(q), \boldsymbol{\theta})) \Big|_{q=t} \right). \tag{9}$$

### 2.2. Preliminaries

We would need the following results from [18]. First, let us extend the fan-beam transform $D_f(\mathbf{a}, \boldsymbol{\theta})$ to $D_f(\mathbf{a}, \mathbf{z})$,

$$D_f(\mathbf{a}, \mathbf{z}) = \int_0^\infty ds f(\mathbf{a} + s\mathbf{z}), \quad (\mathbf{a}, \mathbf{z}) \in \Gamma \times \mathbb{R}^2, \tag{10}$$

which is homogeneous of degree $-1$ in the second argument; that is,

$$\begin{aligned} D_f(\mathbf{a}, r\boldsymbol{\theta}) &= \int_0^\infty ds f(\mathbf{a} + rs\boldsymbol{\theta}) \\ &= r^{-1} \int_0^\infty ds f(\mathbf{a}, s\boldsymbol{\theta}) = r^{-1} D_f(\mathbf{a}, \boldsymbol{\theta}), \quad r > 0. \end{aligned} \tag{11}$$

For a fixed $\mathbf{a} \in \mathbb{R}^2$, let us define a Fourier transform as $\widehat{D_\mathbf{a} f}(\mathbf{v}) = \int_{\mathbb{R}^2} d\mathbf{z} D_f(\mathbf{a}, \mathbf{z}) e^{-i\mathbf{z}\cdot\mathbf{v}}$. This Fourier transform is also homogeneous of degree $-1$, since

$$\begin{aligned} \widehat{D_\mathbf{a} f}(s\boldsymbol{\sigma}) &= \int_{\mathbb{R}^2} d\mathbf{z} D_f(\mathbf{a}, \mathbf{z}) e^{-is\mathbf{z}\cdot\boldsymbol{\sigma}} \\ &= \int_{\mathbb{R}^2} d\mathbf{y} s^{-2} D_f(\mathbf{a}, s^{-1}\mathbf{y}) e^{-i\mathbf{y}\cdot\boldsymbol{\sigma}} \\ &= \int_{\mathbb{R}^2} d\mathbf{y} s^{-1} D_f(\mathbf{a}, \mathbf{y}) e^{-i\mathbf{y}\cdot\boldsymbol{\sigma}} = s^{-1} \widehat{D_\mathbf{a} f}(\boldsymbol{\sigma}). \end{aligned} \tag{12}$$

Hence, we have

$$
\begin{aligned}
\widehat{D_{\mathbf{a}}^{-}f}(\boldsymbol{\sigma}) &= \int_{\mathbb{R}^2} d\mathbf{z} D_f^{-}(\mathbf{a},\mathbf{z}) e^{-i\mathbf{z}\cdot\boldsymbol{\sigma}} \\
&= \int_{\mathbb{R}^2} d\mathbf{z} D_f(\mathbf{a},\mathbf{z}) e^{-i\mathbf{z}\cdot\boldsymbol{\sigma}} - \int_{\mathbb{R}^2} d\mathbf{z} D_f(\mathbf{a},-\mathbf{z}) e^{-i\mathbf{z}\cdot\boldsymbol{\sigma}} \\
&= \int_{\mathbb{R}^2} d\mathbf{z} \int_0^\infty ds f(\mathbf{a}(t)+s\mathbf{z}) e^{-i\mathbf{z}\cdot\boldsymbol{\sigma}} \\
&\quad - \int_{\mathbb{R}^2} d\mathbf{z} \int_0^\infty ds f(\mathbf{a}(t)-s\mathbf{z}) e^{-i\mathbf{z}\cdot\boldsymbol{\sigma}} \\
&= \int_0^\infty ds e^{is^{-1}\mathbf{a}(t)\cdot\boldsymbol{\sigma}} s^{-2} \int_{\mathbb{R}^2} d\mathbf{y} f(\mathbf{y}) e^{-is^{-1}\mathbf{y}\cdot\boldsymbol{\sigma}} \\
&\quad - \int_0^\infty ds e^{-is^{-1}\mathbf{a}(t)\cdot\boldsymbol{\sigma}} s^{-2} \int_{\mathbb{R}^2} d\mathbf{y} f(\mathbf{y}) e^{is^{-1}\mathbf{y}\cdot\boldsymbol{\sigma}} \\
&= \int_0^\infty ds e^{is^{-1}\mathbf{a}(t)\cdot\boldsymbol{\sigma}} s^{-2} \widehat{f}(s^{-1}\boldsymbol{\sigma}) \\
&\quad - \int_0^\infty ds e^{-is^{-1}\mathbf{a}(t)\cdot\boldsymbol{\sigma}} s^{-2} \widehat{f}(-s^{-1}\boldsymbol{\sigma}) \\
&= \int_0^\infty dr e^{ir\mathbf{a}(t)\cdot\boldsymbol{\sigma}} \widehat{f}(r\boldsymbol{\sigma}) - \int_{-\infty}^0 dr e^{ir\mathbf{a}(t)\cdot\boldsymbol{\sigma}} \widehat{f}(r\boldsymbol{\sigma}) \\
&= \int_{-\infty}^\infty dr \operatorname{sgn}(r) e^{ir\mathbf{a}(t)\cdot\boldsymbol{\sigma}} \widehat{f}(r\boldsymbol{\sigma}).
\end{aligned}
\tag{13}
$$

Also, $\widehat{D_{\mathbf{a}}^{-}f}(\boldsymbol{\sigma})$ is an odd function with respect to $\boldsymbol{\sigma}$, that is,

Due to the factor $\delta(\boldsymbol{\xi}\cdot(\mathbf{x}-\mathbf{a}(t)))$, we set $\boldsymbol{\xi}\cdot\mathbf{x} = \boldsymbol{\xi}\cdot\mathbf{a}(t)$ and $\boldsymbol{\theta}^\perp = \pm\boldsymbol{\xi}/\|\boldsymbol{\xi}\|$. Hence, (16) can be simplified as follows:

$$

\begin{aligned}

&PV\int_{t_1}^{t_2} dt \frac{\operatorname{sgn}(\mathbf{e}\cdot\boldsymbol{\theta}^\perp)}{\|\mathbf{x}-\mathbf{a}(t)\|\cdot(\mathbf{a}'(t)\cdot\boldsymbol{\theta}^\perp)} \left( \frac{\partial^2}{\partial q^2} \left(D_f^{+}(\mathbf{a}(q),\theta)\right)\Big|_{q=t} \right) \\

&= \frac{2\pi}{(2\pi)^2} \int_{\mathbb{R}^2} d\boldsymbol{\xi} \widehat{f}(\boldsymbol{\xi}) PV\int_{t_1}^{t_2} dt \frac{\|\boldsymbol{\xi}\| \operatorname{sgn}(\mathbf{e}\cdot\boldsymbol{\xi})}{(\mathbf{a}'(t)\cdot\boldsymbol{\xi})} \\

&\quad \times \left( i\boldsymbol{\xi}\cdot\mathbf{a}''(t) - (\boldsymbol{\xi}\cdot\mathbf{a}'(t))^2 \right) e^{i\boldsymbol{\xi}\cdot\mathbf{x}} \delta(\boldsymbol{\xi}\cdot(\mathbf{x}-\mathbf{a}(t))) \\

&= \frac{2\pi}{(2\pi)^2} \int_{\mathbb{R}^2} d\boldsymbol{\xi} \widehat{f}(\boldsymbol{\xi}) \|\boldsymbol{\xi}\| \operatorname{sgn}(\mathbf{e}\cdot\boldsymbol{\xi}) e^{i\boldsymbol{\xi}\cdot\mathbf{x}} PV\int_{t_1}^{t_2} dt \\

&\quad \times \left( \frac{(i\boldsymbol{\xi}\cdot\mathbf{a}''(t))}{(\mathbf{a}'(t)\cdot\boldsymbol{\xi})} - (\boldsymbol{\xi}\cdot\mathbf{a}'(t)) \right) \delta(\boldsymbol{\xi}\cdot(\mathbf{x}-\mathbf{a}(t))).

\end{aligned}

\tag{17}

$$

On the other hand, we have

$$PV\int_{t_1}^{t_2} dt \frac{\operatorname{sgn}(\mathbf{e}\cdot\boldsymbol{\theta}^{\perp})}{\|\mathbf{x}-\mathbf{a}(t)\|\cdot(\mathbf{a}'(t)\cdot\boldsymbol{\theta}^{\perp})} \times\left(\frac{(\mathbf{a}''(t)\cdot\boldsymbol{\theta}^{\perp})}{(\mathbf{a}'(t)\cdot\boldsymbol{\theta}^{\perp})}\frac{\partial}{\partial q}\left(D_f^{+}(\mathbf{a}(q),\boldsymbol{\theta})\right)\Big|_{q=t}\right)$$

$$= \frac{2\pi}{(2\pi)^2}\int_{\mathbb{R}^2} d\boldsymbol{\xi}\hat{f}(\boldsymbol{\xi})\|\boldsymbol{\xi}\|\operatorname{sgn}(\mathbf{e}\cdot\boldsymbol{\xi})e^{i\boldsymbol{\xi}\cdot\mathbf{x}} \times PV\int_{t_1}^{t_2} dt\frac{(i\boldsymbol{\xi}\cdot\mathbf{a}''(t))}{(\mathbf{a}'(t)\cdot\boldsymbol{\xi})}\delta(\boldsymbol{\xi}\cdot(\mathbf{x}-\mathbf{a}(t))). \tag{18}$$

Utilizing the following relationship:

$$\int_{t_1}^{t_2} dt(\boldsymbol{\xi}\cdot\mathbf{a}'(t))\delta(\boldsymbol{\xi}\cdot(\mathbf{x}-\mathbf{a}(t)))$$

$$= \frac{1}{2\pi}\int_{t_1}^{t_2} dt(\boldsymbol{\xi}\cdot\mathbf{a}'(t))\int_{-\infty}^{\infty} ds e^{is\boldsymbol{\xi}\cdot(\mathbf{x}-\mathbf{a}(t))}$$

$$= \frac{1}{2\pi}\int_{-\infty}^{\infty} ds\int_{t_1}^{t_2} dt(\boldsymbol{\xi}\cdot\mathbf{a}'(t))e^{is\boldsymbol{\xi}\cdot(\mathbf{x}-\mathbf{a}(t))}$$

$$= -\frac{1}{2\pi}\int_{-\infty}^{\infty}\int_{(\mathbf{x}-\mathbf{a}(t_1))\cdot\boldsymbol{\xi}}^{(\mathbf{x}-\mathbf{a}(t_2))\cdot\boldsymbol{\xi}} e^{isu}du\,ds$$

$$= \frac{i}{2\pi}\int_{-\infty}^{\infty}\frac{e^{is(\mathbf{x}-\mathbf{a}(t_2))\cdot\boldsymbol{\xi}}-e^{is(\mathbf{x}-\mathbf{a}(t_1))\cdot\boldsymbol{\xi}}}{s}ds$$

$$= \frac{\operatorname{sgn}((\mathbf{x}-\mathbf{a}(t_1))\cdot\boldsymbol{\xi})-\operatorname{sgn}((\mathbf{x}-\mathbf{a}(t_2))\cdot\boldsymbol{\xi})}{2}$$

$$= \operatorname{sgn}((\mathbf{a}(t_2)-\mathbf{a}(t_1))\cdot\boldsymbol{\xi}) = \operatorname{sgn}(\mathbf{e}\cdot\boldsymbol{\xi}), \tag{19}$$

and subtracting (18) from (17), we obtain

$$PV\int_{t_1}^{t_2} dt\frac{\operatorname{sgn}(\mathbf{e}\cdot\boldsymbol{\theta}^{\perp})}{\|\mathbf{x}-\mathbf{a}(t)\|\cdot(\mathbf{a}'(t)\cdot\boldsymbol{\theta}^{\perp})} \times\left(\frac{\partial^2}{\partial q^2}\left(D_f^{+}(\mathbf{a}(q),\boldsymbol{\theta})\right)\Big|_{q=t} - \frac{(\mathbf{a}''(t)\cdot\boldsymbol{\theta}^{\perp})}{(\mathbf{a}'(t)\cdot\boldsymbol{\theta}^{\perp})}\frac{\partial}{\partial q}\left(D_f^{+}(\mathbf{a}(q),\boldsymbol{\theta})\right)\Big|_{q=t}\right)$$
$$= -\frac{2\pi}{(2\pi)^2}\int_{\mathbb{R}^2} d\boldsymbol{\xi}\hat{f}(\boldsymbol{\xi})\|\boldsymbol{\xi}\|\operatorname{sgn}(\mathbf{e}\cdot\boldsymbol{\xi})e^{i\boldsymbol{\xi}\cdot\mathbf{x}} \times\int_{t_1}^{t_2} dt(\boldsymbol{\xi}\cdot\mathbf{a}'(t))\delta(\boldsymbol{\xi}\cdot(\mathbf{x}-\mathbf{a}(t)))$$
$$= -\frac{2\pi}{(2\pi)^2}\int_{\mathbb{R}^2} d\boldsymbol{\xi}\hat{f}(\boldsymbol{\xi})\|\boldsymbol{\xi}\|\operatorname{sgn}(\mathbf{e}\cdot\boldsymbol{\xi})e^{i\boldsymbol{\xi}\cdot\mathbf{x}}\operatorname{sgn}(\mathbf{e}\cdot\boldsymbol{\xi})$$
$$= -\frac{2\pi}{(2\pi)^2}\int_{\mathbb{R}^2} d\boldsymbol{\xi}\hat{f}(\boldsymbol{\xi})\|\boldsymbol{\xi}\|e^{i\boldsymbol{\xi}\cdot\mathbf{x}} = -2\pi\Lambda f(\mathbf{x}). \tag{20}$$

### 2.4. *Proof of* (9)

For a fixed point $\mathbf{x}_0$ on the chord $L$ from $\mathbf{a}(t_1)$ to $\mathbf{a}(t_2)$, let us define

$$g(\mathbf{y}) = g_1(\mathbf{y}) - g_2(\mathbf{y}), \tag{21}$$

where

$$g_1(\mathbf{y}) = PV\int_{t_1}^{t_2} dt\frac{\operatorname{sgn}(\mathbf{e}\cdot\boldsymbol{\theta}^{\perp}(\mathbf{x}_0,t))}{\|\mathbf{y}-\mathbf{a}(t)\|\cdot(\mathbf{a}'(t)\cdot\boldsymbol{\theta}^{\perp}(\mathbf{x}_0,t))} \times\frac{\partial^2}{\partial q^2}\left(D_f^{-}(\mathbf{a}(q),\boldsymbol{\theta}(\mathbf{y},t))\right)\Big|_{q=t},$$

$$g_2(\mathbf{y}) = PV\int_{t_1}^{t_2} dt\frac{\operatorname{sgn}(\mathbf{e}\cdot\boldsymbol{\theta}^{\perp}(\mathbf{x}_0,t))(\mathbf{a}''(t)\cdot\boldsymbol{\theta}^{\perp}(\mathbf{x}_0,t))}{\|\mathbf{y}-\mathbf{a}(t)\|\cdot(\mathbf{a}'(t)\cdot\boldsymbol{\theta}^{\perp}(\mathbf{x}_0,t))^2} \times\frac{\partial}{\partial q}\left(D_f^{-}(\mathbf{a}(q),\boldsymbol{\theta}(\mathbf{y},t))\right)\Big|_{q=t}. \tag{22}$$

Also, we define an auxiliary 2D Hilbert transform along the direction $\mathbf{e}$ of the chord $L$ as

$$Hg(\mathbf{x}) = \frac{1}{(2\pi)^2 i}\int_{\mathbb{R}^2} d\boldsymbol{\xi}\operatorname{sgn}(\mathbf{e}\cdot\boldsymbol{\xi})\hat{g}(\boldsymbol{\xi})e^{i\mathbf{x}\cdot\boldsymbol{\xi}}, \tag{23}$$

where $\hat{g}(\boldsymbol{\xi})$ is the Fourier transform of $g(\mathbf{y})$. Now, let us evaluate

$$Hg(\mathbf{x}_0) = \frac{1}{(2\pi)^2 i}\int_{\mathbb{R}^2} d\boldsymbol{\xi}\operatorname{sgn}(\mathbf{e}\cdot\boldsymbol{\xi})\hat{g}(\boldsymbol{\xi})e^{i\mathbf{x}_0\cdot\boldsymbol{\xi}} = \frac{1}{(2\pi)^2 i}\int_{\mathbb{R}^2} d\boldsymbol{\xi}\operatorname{sgn}(\mathbf{e}\cdot\boldsymbol{\xi})(\hat{g}_1(\boldsymbol{\xi})-\hat{g}_2(\boldsymbol{\xi}))e^{i\mathbf{x}_0\cdot\boldsymbol{\xi}}, \tag{24}$$

where $\hat{g}_1(\boldsymbol{\xi})$ and $\hat{g}_2(\boldsymbol{\xi})$ are the Fourier transforms of $g_1(\mathbf{y})$ and $g_2(\mathbf{y})$, respectively. Note that

$$\hat{g}_1(\boldsymbol{\xi}) = \int_{\mathbb{R}^2} d\mathbf{y}e^{-i\mathbf{y}\cdot\boldsymbol{\xi}}PV\int_{t_1}^{t_2} dt\frac{\operatorname{sgn}(\mathbf{e}\cdot\boldsymbol{\theta}^{\perp}(\mathbf{x}_0,t))}{\|\mathbf{y}-\mathbf{a}(t)\|\cdot(\mathbf{a}'(t)\cdot\boldsymbol{\theta}^{\perp}(\mathbf{x}_0,t))} \times\frac{\partial^2}{\partial q^2}\left(D_f^{-}(\mathbf{a}(q),\boldsymbol{\theta}(\mathbf{y},t))\right)\Big|_{q=t}$$

$$= PV\int_{t_1}^{t_2} dt\frac{\operatorname{sgn}(\mathbf{e}\cdot\boldsymbol{\theta}^{\perp}(\mathbf{x}_0,t))}{(\mathbf{a}'(t)\cdot\boldsymbol{\theta}^{\perp}(\mathbf{x}_0,t))} \times\frac{\partial^2}{\partial q^2}\int_{\mathbb{R}^2} d\mathbf{y}e^{-i\mathbf{y}\cdot\boldsymbol{\xi}}\left(D_f^{-}(\mathbf{a}(q),\mathbf{y}-\mathbf{a}(t))\right)\Big|_{q=t}$$

$$= PV\int_{t_1}^{t_2} dt\frac{\operatorname{sgn}(\mathbf{e}\cdot\boldsymbol{\theta}^{\perp}(\mathbf{x}_0,t))}{(\mathbf{a}'(t)\cdot\boldsymbol{\theta}^{\perp}(\mathbf{x}_0,t))}e^{-i\mathbf{a}(t)\cdot\boldsymbol{\xi}} \times\frac{\partial^2}{\partial q^2}\int_{\mathbb{R}^2} d\mathbf{z}e^{-i\mathbf{z}\cdot\boldsymbol{\xi}}\left(D_f^{-}(\mathbf{a}(q),\mathbf{z})\right)\Big|_{q=t}$$

$$= PV\int_{t_1}^{t_2} dt\frac{\operatorname{sgn}(\mathbf{e}\cdot\boldsymbol{\theta}^{\perp}(\mathbf{x}_0,t))}{(\mathbf{a}'(t)\cdot\boldsymbol{\theta}^{\perp}(\mathbf{x}_0,t))}e^{-i\mathbf{a}(t)\cdot\boldsymbol{\xi}}\frac{\partial^2}{\partial t^2}\widehat{D_{\mathbf{a}(t)}^{-}f}(\boldsymbol{\xi}). \tag{25}$$

Letting $\boldsymbol{\xi} = s\boldsymbol{\sigma}$, we have

$$\begin{aligned}

&\frac{1}{(2\pi)^2 i}\int_{\mathbb{R}^2} d\boldsymbol{\xi}\,\mathrm{sgn}(\mathbf{e}\cdot\boldsymbol{\xi})\hat{g}_1(\boldsymbol{\xi})e^{i\mathbf{x}_0\cdot\boldsymbol{\xi}}\\

&\quad=\frac{1}{(2\pi)^2 i}\int_{\mathbb{R}^2} d\boldsymbol{\xi}\,\mathrm{sgn}(\mathbf{e}\cdot\boldsymbol{\xi})e^{i\mathbf{x}_0\cdot\boldsymbol{\xi}}\\

&\qquad\times PV\int_{t_1}^{t_2} dt\frac{\mathrm{sgn}\left(\mathbf{e}\cdot\boldsymbol{\theta}^{\perp}(\mathbf{x}_0,t)\right)}{\left(\mathbf{a}'(t)\cdot\boldsymbol{\theta}^{\perp}(\mathbf{x}_0,t)\right)}e^{-i\mathbf{a}(t)\cdot\boldsymbol{\xi}}\frac{\partial^2}{\partial t^2}\widehat{D^{-}_{\mathbf{a}(t)}f}(\boldsymbol{\xi})\\

&\quad=\frac{1}{(2\pi)^2 i}\int_{\mathbb{S}} d\boldsymbol{\sigma}\int_0^{\infty} s\,ds\,\mathrm{sgn}(\mathbf{e}\cdot\boldsymbol{\sigma})e^{is\mathbf{x}_0\cdot\boldsymbol{\sigma}}\frac{\partial^2}{\partial t^2}\widehat{D^{-}_{\mathbf{a}(t)}f}(s\boldsymbol{\sigma})\\

&\qquad\times PV\int_{t_1}^{t_2} dt\frac{\mathrm{sgn}\left(\mathbf{e}\cdot\boldsymbol{\theta}^{\perp}(\mathbf{x}_0,t)\right)}{\left(\mathbf{a}'(t)\cdot\boldsymbol{\theta}^{\perp}(\mathbf{x}_0,t)\right)}e^{-is\mathbf{a}(t)\cdot\boldsymbol{\sigma}}\\

&\quad=\frac{1}{(2\pi)^2 i}\int_{\mathbb{S}} d\boldsymbol{\sigma}\int_0^{\infty} ds\,\mathrm{sgn}(\mathbf{e}\cdot\boldsymbol{\sigma})e^{is\mathbf{x}_0\cdot\boldsymbol{\sigma}}\\

&\qquad\times PV\int_{t_1}^{t_2} dt\frac{\mathrm{sgn}\left(\mathbf{e}\cdot\boldsymbol{\theta}^{\perp}(\mathbf{x}_0,t)\right)}{\left(\mathbf{a}'(t)\cdot\boldsymbol{\theta}^{\perp}(\mathbf{x}_0,t)\right)}e^{-is\mathbf{a}(t)\cdot\boldsymbol{\sigma}}\frac{\partial^2}{\partial t^2}\widehat{D^{-}_{\mathbf{a}(t)}f}(\boldsymbol{\sigma})\\

&\quad=\frac{1}{(2\pi)^2 i}\int_{\mathbb{S}} d\boldsymbol{\sigma}\,\mathrm{sgn}(\mathbf{e}\cdot\boldsymbol{\sigma})PV\int_{t_1}^{t_2} dt\frac{\mathrm{sgn}\left(\mathbf{e}\cdot\boldsymbol{\theta}^{\perp}(\mathbf{x}_0,t)\right)}{\left(\mathbf{a}'(t)\cdot\boldsymbol{\theta}^{\perp}(\mathbf{x}_0,t)\right)}\\

&\qquad\times\frac{\partial^2}{\partial t^2}\widehat{D^{-}_{\mathbf{a}(t)}f}(\boldsymbol{\sigma})\int_0^{\infty} ds e^{is(\mathbf{x}_0-\mathbf{a}(t))\cdot\boldsymbol{\sigma}}.

\end{aligned}\tag{26}$$

Since $\int_{\mathbb{S}} d\boldsymbol{\sigma}h(\boldsymbol{\sigma}) = 0$, if $h(\boldsymbol{\sigma})$ is an odd function with respect to $\boldsymbol{\sigma}$, (26) can be simplified as

$$\begin{aligned}

&\frac{1}{(2\pi)^2 i}\int_{\mathbb{S}} d\boldsymbol{\sigma}\,\mathrm{sgn}(\mathbf{e}\cdot\boldsymbol{\sigma})PV\int_{t_1}^{t_2} dt\frac{\mathrm{sgn}\left(\mathbf{e}\cdot\boldsymbol{\theta}^{\perp}(\mathbf{x}_0,t)\right)}{\left(\mathbf{a}'(t)\cdot\boldsymbol{\theta}^{\perp}(\mathbf{x}_0,t)\right)}\\

&\qquad\times\frac{\partial^2}{\partial t^2}\widehat{D^{-}_{\mathbf{a}(t)}f}(\boldsymbol{\sigma})\int_0^{\infty} ds e^{is(\mathbf{x}_0-\mathbf{a}(t))\cdot\boldsymbol{\sigma}}\\

&\quad=\frac{1}{(2\pi)^2 i}\int_{\mathbb{S}} d\boldsymbol{\sigma}\,\mathrm{sgn}(\mathbf{e}\cdot\boldsymbol{\sigma})PV\int_{t_1}^{t_2} dt\frac{\mathrm{sgn}\left(\mathbf{e}\cdot\boldsymbol{\theta}^{\perp}(\mathbf{x}_0,t)\right)}{\left(\mathbf{a}'(t)\cdot\boldsymbol{\theta}^{\perp}(\mathbf{x}_0,t)\right)}\\

&\qquad\times\frac{\partial^2}{\partial t^2}\widehat{D^{-}_{\mathbf{a}(t)}f}(\boldsymbol{\sigma})\int_{-\infty}^{\infty}\left(\frac{1+\mathrm{sgn}(s)}{2}\right)ds e^{is(\mathbf{x}_0-\mathbf{a}(t))\cdot\boldsymbol{\sigma}}\\

&\quad=\frac{1}{2(2\pi)^2 i}\int_{\mathbb{S}} d\boldsymbol{\sigma}\,\mathrm{sgn}(\mathbf{e}\cdot\boldsymbol{\sigma})PV\int_{t_1}^{t_2} dt\frac{\mathrm{sgn}\left(\mathbf{e}\cdot\boldsymbol{\theta}^{\perp}(\mathbf{x}_0,t)\right)}{\left(\mathbf{a}'(t)\cdot\boldsymbol{\theta}^{\perp}(\mathbf{x}_0,t)\right)}\\

&\qquad\times\frac{\partial^2}{\partial t^2}\widehat{D^{-}_{\mathbf{a}(t)}f}(\boldsymbol{\sigma})\int_{-\infty}^{\infty} ds e^{is(\mathbf{x}_0-\mathbf{a}(t))\cdot\boldsymbol{\sigma}}\\

&\quad=\frac{2\pi}{2(2\pi)^2 i}\int_{\mathbb{S}} d\boldsymbol{\sigma}\,\mathrm{sgn}(\mathbf{e}\cdot\boldsymbol{\sigma})PV\int_{t_1}^{t_2} dt\frac{\mathrm{sgn}\left(\mathbf{e}\cdot\boldsymbol{\theta}^{\perp}(\mathbf{x}_0,t)\right)}{\left(\mathbf{a}'(t)\cdot\boldsymbol{\theta}^{\perp}(\mathbf{x}_0,t)\right)}\\

&\qquad\times\frac{\partial^2}{\partial t^2}\widehat{D^{-}_{\mathbf{a}(t)}f}(\boldsymbol{\sigma})\delta\left((\mathbf{x}_0-\mathbf{a}(t))\cdot\boldsymbol{\sigma}\right)\\

&\quad=\frac{2\pi}{2(2\pi)^2 i}\int_{\mathbb{S}} d\boldsymbol{\sigma}\,\mathrm{sgn}(\mathbf{e}\cdot\boldsymbol{\sigma})PV\int_{t_1}^{t_2} dt\frac{\mathrm{sgn}\left(\mathbf{e}\cdot\boldsymbol{\theta}^{\perp}(\mathbf{x}_0,t)\right)}{\left(\mathbf{a}'(t)\cdot\boldsymbol{\theta}^{\perp}(\mathbf{x}_0,t)\right)}\\

&\qquad\times\frac{\partial^2}{\partial t^2}\left(\int_{-\infty}^{\infty} dr\,\mathrm{sgn}(r)e^{ir\mathbf{a}(t)\cdot\boldsymbol{\sigma}}\hat{f}(r\boldsymbol{\sigma})\right)\delta\left((\mathbf{x}_0-\mathbf{a}(t))\cdot\boldsymbol{\sigma}\right)\\

&\quad=\frac{2\pi}{2(2\pi)^2 i}\int_{\mathbb{S}} d\boldsymbol{\sigma}\,\mathrm{sgn}(\mathbf{e}\cdot\boldsymbol{\sigma})PV\int_{t_1}^{t_2} dt\frac{\mathrm{sgn}\left(\mathbf{e}\cdot\boldsymbol{\theta}^{\perp}(\mathbf{x}_0,t)\right)}{\left(\mathbf{a}'(t)\cdot\boldsymbol{\theta}^{\perp}(\mathbf{x}_0,t)\right)}\\

&\qquad\times\left(\int_{-\infty}^{\infty} dr\,\mathrm{sgn}(r)\left(ir\mathbf{a}''(t)\cdot\boldsymbol{\sigma}-\left(r\mathbf{a}'(t)\cdot\boldsymbol{\sigma}\right)^2\right)\right.\\

&\qquad\qquad\left.\times e^{ir\mathbf{a}(t)\cdot\boldsymbol{\sigma}}\hat{f}(r\boldsymbol{\sigma})\right)\delta\left((\mathbf{x}_0-\mathbf{a}(t))\cdot\boldsymbol{\sigma}\right)\\

&\quad=\frac{2\pi}{2(2\pi)^2 i}\int_{\mathbb{S}} d\boldsymbol{\sigma}\,\mathrm{sgn}(\mathbf{e}\cdot\boldsymbol{\sigma})PV\int_{t_1}^{t_2} dt\frac{\mathrm{sgn}(\mathbf{e}\cdot\boldsymbol{\sigma})}{(\mathbf{a}'(t)\cdot\boldsymbol{\sigma})}\\

&\qquad\times\left(\int_{-\infty}^{\infty} dr\,\mathrm{sgn}(r)\left(ir\mathbf{a}''(t)\cdot\boldsymbol{\sigma}-\left(r\mathbf{a}'(t)\cdot\boldsymbol{\sigma}\right)^2\right)\right.\\

&\qquad\qquad\left.\times e^{ir\mathbf{x}_0\cdot\boldsymbol{\sigma}}\hat{f}(r\boldsymbol{\sigma})\right)\delta\left((\mathbf{x}_0-\mathbf{a}(t))\cdot\boldsymbol{\sigma}\right)\\

&\quad=\frac{2\pi}{2(2\pi)^2 i}\int_{\mathbb{S}} d\boldsymbol{\sigma}PV\int_{t_1}^{t_2} dt\\

&\qquad\times\left(\int_{-\infty}^{\infty} dr\,\mathrm{sgn}(r)e^{ir\mathbf{x}_0\cdot\boldsymbol{\sigma}}\right.\\

&\qquad\qquad\left.\times\left(\frac{ir\mathbf{a}''(t)\cdot\boldsymbol{\sigma}}{\mathbf{a}'(t)\cdot\boldsymbol{\sigma}}-r^2\left(\mathbf{a}'(t)\cdot\boldsymbol{\sigma}\right)\right)\hat{f}(r\boldsymbol{\sigma})\right)\\

&\qquad\times\delta\left((\mathbf{x}_0-\mathbf{a}(t))\cdot\boldsymbol{\sigma}\right).

\end{aligned}\tag{27}$$

Again, due to the factor $\delta((\mathbf{x}_0-\mathbf{a}(t))\cdot\boldsymbol{\sigma})$, we set $\mathbf{a}(t)\cdot\boldsymbol{\sigma} = \mathbf{x}_0\cdot\boldsymbol{\sigma}$ and $\boldsymbol{\theta}^{\perp}(\mathbf{x}_0,t) = \pm\boldsymbol{\sigma}$ in (27). On the other hand, we have

$$\begin{aligned}

&\frac{1}{(2\pi)^2 i}\int_{\mathbb{R}^2} d\boldsymbol{\xi}\,\mathrm{sgn}(\mathbf{e}\cdot\boldsymbol{\xi})\hat{g}_2(\boldsymbol{\xi})e^{i\mathbf{x}_0\cdot\boldsymbol{\xi}}\\

&\quad=\frac{2\pi}{2(2\pi)^2 i}\int_{\mathbb{S}} d\boldsymbol{\sigma}PV\int_{t_1}^{t_2} dt\\

&\qquad\times\left(\int_{-\infty}^{\infty} dr\,\mathrm{sgn}(r)e^{ir\mathbf{x}_0\cdot\boldsymbol{\sigma}}\left(\frac{ir\mathbf{a}''(t)\cdot\boldsymbol{\sigma}}{\mathbf{a}'(t)\cdot\boldsymbol{\sigma}}\right)\hat{f}(r\boldsymbol{\sigma})\right)\\

&\qquad\times\delta\left((\mathbf{x}_0-\mathbf{a}(t))\cdot\boldsymbol{\sigma}\right).

\end{aligned}\tag{28}$$

Combining (27) and (28), we obtain

$$\begin{aligned}

Hg(\mathbf{x}_0)&=\frac{1}{(2\pi)^2 i}\int_{\mathbb{R}^2} d\boldsymbol{\xi}\,\mathrm{sgn}(\mathbf{e}\cdot\boldsymbol{\xi})\hat{g}(\boldsymbol{\xi})e^{i\mathbf{x}_0\cdot\boldsymbol{\xi}}\\

&=\frac{2\pi}{2(2\pi)^2 i}\int_{\mathbb{S}} d\boldsymbol{\sigma}\int_{t_1}^{t_2} dt\\

&\quad\times\left(\int_{-\infty}^{\infty} dr\,\mathrm{sgn}(r)e^{ir\mathbf{x}_0\cdot\boldsymbol{\sigma}}\left(-r^2\left(\mathbf{a}'(t)\cdot\boldsymbol{\sigma}\right)\right)\hat{f}(r\boldsymbol{\sigma})\right)\\

&\quad\times\delta\left((\mathbf{x}_0-\mathbf{a}(t)\cdot\boldsymbol{\sigma})\right)\\

&=-\frac{2\pi}{2(2\pi)^2 i}\int_{\mathbb{S}} d\boldsymbol{\sigma}\int_{-\infty}^{\infty} dr\,\mathrm{sgn}(r)e^{ir\mathbf{x}_0\cdot\boldsymbol{\sigma}}r^2\hat{f}(r\boldsymbol{\sigma})\int_{t_1}^{t_2} dt\\

&\quad\times\left(\mathbf{a}'(t)\cdot\boldsymbol{\sigma}\right)\delta\left((\mathbf{x}_0-\mathbf{a}(t))\cdot\boldsymbol{\sigma}\right)\\

&=-\frac{2\pi}{2(2\pi)^2 i}\int_{\mathbb{S}} d\boldsymbol{\sigma}\int_{-\infty}^{\infty} dr\,\mathrm{sgn}(r)r^2\hat{f}(r\boldsymbol{\sigma})e^{ir\mathbf{x}_0\cdot\boldsymbol{\sigma}}\\

&\quad\times\mathrm{sgn}(\mathbf{e}\cdot\boldsymbol{\sigma}),

\end{aligned}\tag{29}$$

where the last step is based on the result from (19).

Noting that $\int_{\mathbb{S}} d\boldsymbol{\sigma} h(-\sigma) = \int_{\mathbb{S}} d\boldsymbol{\sigma} h(\boldsymbol{\sigma})$ for any function $h(\boldsymbol{\sigma})$, we have

$$

\begin{aligned}

&-\frac{2\pi}{2(2\pi)^2 i}\int_{\mathbb{S}} d\boldsymbol{\sigma}\int_{-\infty}^{0} dr\, \mathrm{sgn}(r) r^2 \hat{f}(r\boldsymbol{\sigma}) e^{ir\mathbf{x}_0\cdot\boldsymbol{\sigma}}\, \mathrm{sgn}(\mathbf{e}\cdot\boldsymbol{\sigma})\\

&\quad = -\frac{2\pi}{2(2\pi)^2 i}\int_{\mathbb{S}} d\boldsymbol{\sigma}\int_{-\infty}^{0} dr\, \mathrm{sgn}(r) r^2 \hat{f}(-r\boldsymbol{\sigma}) e^{-ir\mathbf{x}_0\cdot\boldsymbol{\sigma}}\\

&\qquad \times \mathrm{sgn}(-\mathbf{e}\cdot\boldsymbol{\sigma})\\

&\quad = -\frac{2\pi}{2(2\pi)^2 i}\int_{\mathbb{S}} d\boldsymbol{\sigma}\int_{0}^{\infty} dr\, \mathrm{sgn}(r) r^2 \hat{f}(r\boldsymbol{\sigma}) e^{ir\mathbf{x}_0\cdot\boldsymbol{\sigma}}\\

&\qquad \times \mathrm{sgn}(\mathbf{e}\cdot\boldsymbol{\sigma}),

\end{aligned}

\tag{30}

$$

$$
\begin{aligned}
Hg(\mathbf{x}_0) &= \frac{1}{(2\pi)^2 i}\int_{\mathbb{R}^2} d\boldsymbol{\xi}\, \mathrm{sgn}(\mathbf{e}\cdot\boldsymbol{\xi})\hat{g}(\boldsymbol{\xi}) e^{i\mathbf{x}_0\cdot\boldsymbol{\xi}}\\
&= -\frac{2\pi}{(2\pi)^2 i}\int_{\mathbb{S}} d\boldsymbol{\sigma}\int_{0}^{\infty} dr\, \mathrm{sgn}(r) r^2 \hat{f}(r\boldsymbol{\sigma}) e^{ir\mathbf{x}_0\cdot\boldsymbol{\sigma}}\\
&\quad \times \mathrm{sgn}(\mathbf{e}\cdot\boldsymbol{\sigma})\\
&= -\frac{2\pi}{(2\pi)^2 i}\int_{\mathbb{R}^2} d\boldsymbol{\xi}\, \mathrm{sgn}(\mathbf{e}\cdot\boldsymbol{\xi}) \|\boldsymbol{\xi}\| \hat{f}(\boldsymbol{\xi}) e^{i\mathbf{x}_0\cdot\boldsymbol{\xi}}\\
&= \frac{1}{(2\pi)^2 i}\int_{\mathbb{R}^2} d\boldsymbol{\xi}\, \mathrm{sgn}(\mathbf{e}\cdot\boldsymbol{\xi})\left(-2\pi\|\boldsymbol{\xi}\|\hat{f}(\boldsymbol{\xi})\right) e^{i\mathbf{x}_0\cdot\boldsymbol{\xi}}.
\end{aligned}
\tag{31}
$$

From (31), we can conclude that

$$
\hat{g}(\boldsymbol{\xi}) = -2\pi\hat{f}(\boldsymbol{\xi})\|\boldsymbol{\xi}\|. \tag{32}
$$

By the inverse Fourier transform, we obtain

$$
g(\mathbf{x}_0) = -\frac{2\pi}{(2\pi)^2}\int_{\mathbb{R}^2} d\boldsymbol{\xi}\|\boldsymbol{\xi}\|\hat{f}(\boldsymbol{\xi}) e^{i\mathbf{x}_0\cdot\boldsymbol{\xi}} = -2\pi\Lambda f(\mathbf{x}_0). \tag{33}
$$

### 3. IMPLEMENTATION

Without loss of generality, we describe a scanning locus as follows:

$$
\mathbf{a}(t) = \left(R(t)\sin(t), R(t)\cos(t)\right), \tag{34}
$$

where $t$ is the rotational angle about the natural coordinate system origin, and $R(t)$ the variable radius. As shown in Figure 2, equispatial data are collected in our simulation. Denoting $\mathbf{E}_u = (-\sin(t), \cos(t))$ and $\mathbf{E}_w = (-\cos(t), -\sin(t))$, we can form a local coordinate system with fan-beam data measured on a collinear detector array along $\mathbf{E}_u$ at a distance $D_d(t) = R(t) + D_c$, where $D_c$ is a constant. Letting a signed distance $u$ along the direction $\mathbf{E}_u$ be the detector coordinate, and letting $u = 0$ correspond to the orthogonal projection of $\mathbf{a}(t)$ for any fixed $\boldsymbol{\theta}$, we can compute the projection position as

$$
u = \frac{D_d(t)\boldsymbol{\theta}\cdot\mathbf{E}_u}{\boldsymbol{\theta}\cdot\mathbf{E}_w}. \tag{35}
$$

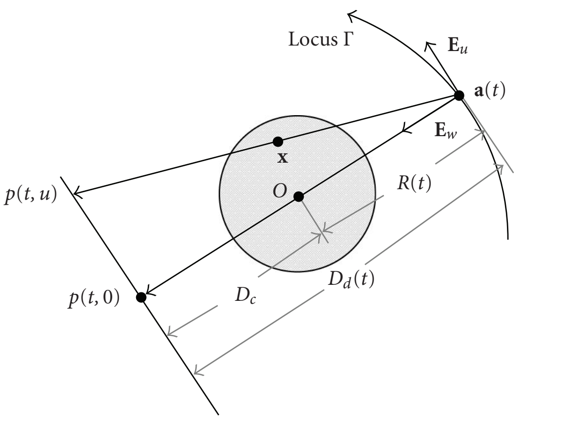

Figure 2: Local coordinate system for a collinear detection along a general planar scanning trajectory.

Finally, let $p(t,u) \equiv D_f(\mathbf{a}(t), \boldsymbol{\theta})$ represent the measured projection data. Using the derivative chain rules, we have

$$
\left.\frac{dp(t,u)}{dt}\right|_{\boldsymbol{\theta}\text{ fixed}} = \left(\frac{\partial}{\partial t} + \frac{\partial u}{\partial t}\frac{\partial}{\partial u}\right)p(t,u), \tag{36}
$$

$$

\begin{aligned}

&\left.\frac{d^2 p(t,u)}{dt^2}\right|_{\boldsymbol{\theta}\text{ fixed}}\\

&\quad = \left(\frac{\partial^2}{\partial^2 t} + \left(\frac{\partial u}{\partial t}\right)^2\frac{\partial^2}{\partial u^2} + \frac{2\partial u}{\partial t}\frac{\partial^2}{\partial t\partial u} + \frac{\partial^2 u}{\partial t^2}\frac{\partial}{\partial u}\right)p(t,u),

\end{aligned}

\tag{37}

$$

$$

\frac{\partial u}{\partial t} = \frac{D_d'(t)u + u^2 + D_d^2(t)}{D_d(t)}, \tag{38}

$$

$$

\frac{\partial^2 u}{\partial t^2} = \frac{D_d(t)D_d''(t)u + 2\left(D_d^2(t) + u^2\right)\left(D_d'(t) + u\right)}{D_d^2(t)}, \tag{39}

$$

where $D_d'(t) = dD_d(t)/dt = dR(t)/dt$ and $D_d''(t) = d^2 D_d(t)/dt^2 = d^2R(t)/dt^2$.

Noting that $\boldsymbol{\theta}^{\perp}(\mathbf{x},t) = (-\theta_2, \theta_1)$ or $(\theta_2, -\theta_1)$ if $\boldsymbol{\theta}(\mathbf{x},t) = (\theta_1, \theta_2)$, we can implement our LT algorithm in the steps shown in Algorithm 1.

### 4. SIMULATION

To test the proposed formula, we implemented it in Matlab on a PC (1.0 Gagabyte memory, 2.8 GHz CPU), with all the computationally intensive parts coded in C. In our simulation, we selected an elliptical scanning locus with $R(t) = R_aR_b/\sqrt{R_b\cos^2(t) + R_a\sin^2(t)}$, where $R_a = 40$ and $R_b = 50$ cm. We set the distance $D_c$ to 45 cm, and the detector aperture length 0.1 cm. For a complete scanning turn, we equiangularly collected 720 projections. Also, we assumed that the detector was always centered at the system origin. Since there were numerous chords through any fixed point $\mathbf{x}$, as shown in Figure 3, we selected the one through the origin $o$ and $\mathbf{x}$.

Table 1: Parameters of the DSLP.

| No. | $a$ | $b$ | $c$ | $x_{01}$ | $x_{02}$ | $x_{03}$ | $\varphi$ | $\mu$ | $m$ | $n$ | $\kappa$ |
|---|---|---|---|---|---|---|---|---|---|---|---|
| 1 | 6.900 | 9.00 | 9.00 | 0 | 0 | 0 | 0 | 2.0 | 20 | 40 | 0.98 |
| 2 | 6.792 | 8.82 | 8.82 | 0 | 0 | 0 | 0 | −0.98 | 20 | 40 | 0.98 |
| 3 | 4.100 | 1.60 | 2.10 | −2.2 | 0 | −2.5 | 108 | −0.02 | 3 | 6 | 0.90 |
| 4 | 3.100 | 1.10 | 2.20 | 2.2 | 0 | −2.5 | 72 | −0.02 | 3 | 6 | 0.90 |
| 5 | 2.100 | 2.50 | 5.00 | 0 | 3.5 | −2.5 | 0 | 0.02 | 3 | 6 | 0.90 |
| 6 | 0.460 | 0.46 | 0.46 | 0 | 1.0 | −2.5 | 0 | 0.02 | 2 | 4 | 0.80 |
| 7 | 0.460 | 0.23 | 0.20 | −0.8 | −6.5 | −2.5 | 0 | 0.01 | 2 | 4 | 0.80 |
| 8 | 0.460 | 0.23 | 0.20 | 0.6 | −6.5 | −2.5 | 90 | 0.01 | 2 | 4 | 0.80 |
| 9 | 0.560 | 0.40 | 1.00 | 0.6 | −1.05 | 6.25 | 90 | 0.02 | 2 | 4 | 0.80 |
| 10 | 0.560 | 0.56 | 1.00 | 0 | 1.0 | 6.25 | 0 | −0.02 | 2 | 4 | 0.80 |
| 11 | 20.00 | 15.0 | 500 | 50.0 | 40.0 | 0 | 0 | 0.5 | 1 | 2 | 0.10 |

*Step 1.* For every $t$, compute $dp(t,u)/dt$ and $d^2p(t,u)/dt^2$ according to (36) and (37).

*Step 2.* For every $\mathbf{x}$ inside an ROI.
*Step 2.1.* Determine a pair of parameters $(t_1, t_2)$ such that $\mathbf{x}$, $\mathbf{a}(t_1)$ and $\mathbf{a}(t_2)$ are collinear.
*Step 2.2.* Reconstruct $\Lambda f(\mathbf{x})$ as follows:

$$\Lambda f(\mathbf{x}) = \frac{1}{2\pi}\int_{t_1}^{t_2} dt \frac{\operatorname{sgn}(\mathbf{e}\cdot\boldsymbol{\theta}^{\perp})}{\|\mathbf{x}-\mathbf{a}(t)\|\cdot(\mathbf{a}'(t)\cdot\boldsymbol{\theta}^{\perp})} \times \left(\frac{d^2p(t,u^*)}{dt^2} - \frac{(\mathbf{a}''(t)\cdot\boldsymbol{\theta}^{\perp})}{(\mathbf{a}'(t)\cdot\boldsymbol{\theta}^{\perp})}\frac{dp(t,u^*)}{dt}\right)dt,$$

where

$$u^* = \frac{D_d(t)(\mathbf{x}-\mathbf{a}(t))\cdot\mathbf{E}_u}{(\mathbf{x}-\mathbf{a}(t))\cdot\mathbf{E}_w}.$$

Algorithm 1: Implementation of LT algorithm.

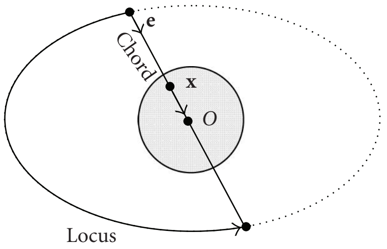

Figure 3: Selection of a chord through both $\mathbf{x}$ and the system origin.

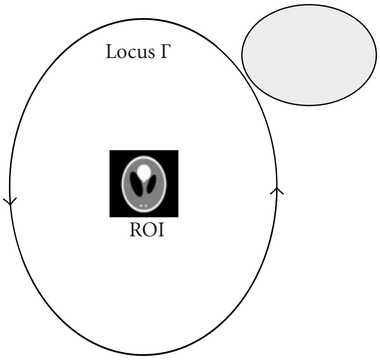

Figure 4: Exact fan-beam lambda tomography with an object outside the scanning trajectory.

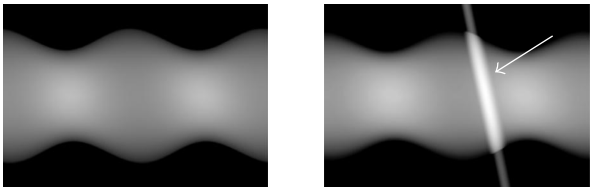

(a) (b)

Figure 5: Sinograms formed with a 50 cm length detector by orbiting the source for one elliptical turn as shown in Figure 2. (a) Sinogram without the outside ellipsoid, (b) the counterpart sinogram with the outside ellipsoid. The oblique strip pointed by the white arrow was due to the outside ellipsoid.

The reconstructed object is the 2D slice at $x_{03} = -2.5$ cm of the 3D differentiable Shepp-Logan phantom (DSLP) [21]. Here the DSLP includes a set of smooth ellipsoids whose parameters are listed in Table 1, where $a$, $b$, $c$ represent the $x_1$, $x_2$, $x_3$ semiaxes, $(x_{01}, x_{02}, x_{03})$ the center of the ellipsoid, $\varphi$ denotes the rotation angle (about $x_3$-axis), $\mu$ the relative attenuation coefficient, $m$, $n$, and $\kappa$ are unsharpening parameters defined in [21]. The unit for $a$, $b$, $c$, and $(x_{01}, x_{02}, x_{03})$ is cm.

Theorem 1 implies that structures outside a scanning trajectory do not affect the exact reconstruction of points inside the trajectory even if the data may be measured through the outside features. To illustrate this property, we simulated with the phantom in two variants. In the first case, the phantom only included the first ten ellipsoids all strictly inside the scanning locus. In the second case, we used all the 11 ellipsoids with the 11th ellipsoid outside the scanning locus, as shown in Figure 4. As a result, the 11th ellipsoid exhibited

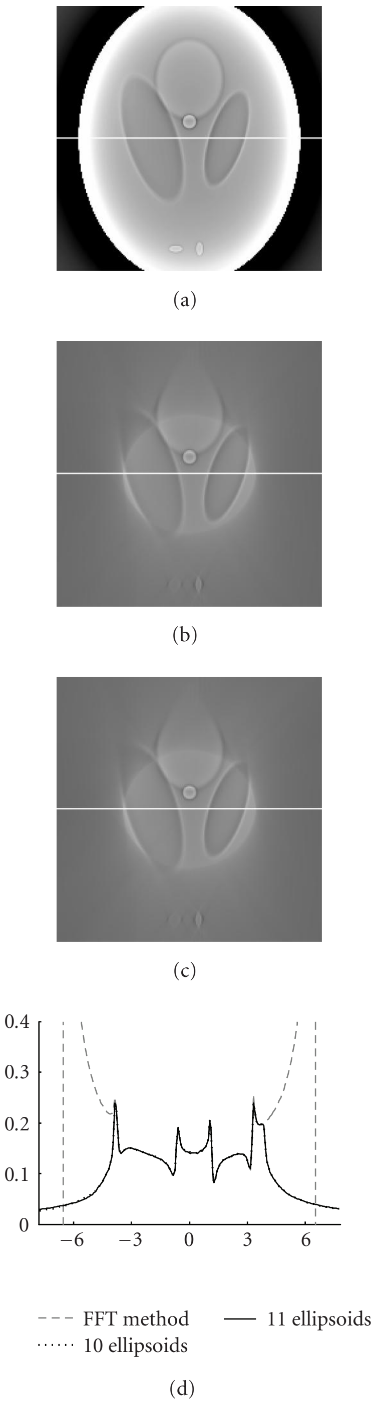

Figure 6: Reconstructed LT images assuming a 15 cm length detector with a display window $[-0.3, 0.5]$. (a) The ground truth reconstructed using the FFT directly from the ideal phantom slice, (b) and (c) the LT images reconstructed from the data without and with the 11th ellipsoid outside the scanning locus, respectively, and (d) representative profiles along the white lines in (a), (b), and (c).

itself as an oblique strip in the sinogram when the X-ray source was orbited for one turn, as shown in Figure 5.

For different detector sizes, various ROI images can be reconstructed. In our simulation, a 15 cm length detector was used. The reconstructed images of $\Lambda f(\mathbf{x})$ with and without the 11th ellipsoid are in Figure 6. As the ground truth, we computed the ideal image $\Lambda f(\mathbf{x})$ from the phantom image $f(\mathbf{x})$ by its definition (2) using FFT. Compared to the ideal image, it was observed that the LT images in the ROI were indeed accurately recovered whether or not there was the 11th ellipsoid in the imaging process.

### 5. DISCUSSION AND CONCLUSION

While the object to be reconstructed is usually restricted within the scanning trajectory, this restriction cannot be always satisfied in the field of biomedical imaging, such as in some PET/SPECT studies, and so on. As demonstrated in Figure 4, Theorem 1 allows that an LT image can be exactly reconstructed even if there are other components outside the trajectory. This property gives us some freedom in designing the imaging geometry and protocols.

In conclusion, we have proved the first exact and efficient general fan-beam LT formula based on the even and odd data extensions. The numerical simulation has verified the correctness of the formulation. The same idea can be extended to the cone-beam geometry with a general scanning trajectory, on which we are actively working. Relevant results will be published in the future.

### ACKNOWLEDGMENTS

The authors are grateful for anonymous reviewers whose suggestions led to this well-integrated paper by combining the two shorter submissions on fan-beam LT of the authors, which are based on the even and old extensions, respectively. The authors also thank Drs. Yangbo Ye and Yuchuan Wei for extensive discussions. This work is partially supported by NIH/NIBIB Grants (EB002667 and EB00428).

### REFERENCES

[1] F. Natterer, *The Mathematics of Computerized Tomography*, John Wiley & Sons, New York, NY, USA, 1986.

[2] M. A. Anastasio, D. Shi, X. Pan, C. Pelizzari, and P. Munro, "A preliminary investigation of local tomography for megavoltage CT imaging," *Medical Physics*, vol. 30, no. 11, pp. 2969–2980, 2003.

[3] A. Faridani, K. Buglione, P. Huabsomboon, O. D. Iancu, and J. McGrath, "Introduction to local tomography," in *Radon Transforms and Tomography*, pp. 29–47, American Mathematical Society, Providence, RI, USA, 2001.

[4] A. Faridani, D. V. Finch, E. L. Ritman, and K. T. Smith, "Local tomography II," *SIAM Journal on Applied Mathematics*, vol. 57, no. 4, pp. 1095–1127, 1997.

[5] A. Faridani, E. L. Ritman, and K. T. Smith, "Local tomography," *SIAM Journal on Applied Mathematics*, vol. 52, no. 2, pp. 459–484, 1992.

[6] A. Katsevich, "Cone beam local tomography," *SIAM Journal on Applied Mathematics*, vol. 59, no. 6, pp. 2224–2246, 1999.

[7] A. K. Louis and P. Maass, "Contour reconstruction in 3-D X-ray CT," *IEEE Transactions on Medical Imaging*, vol. 12, no. 4, pp. 764–769, 1993.

[8] A. G. Ramm and A. Katsevich, *The Radon Transform and Local Tomography*, CRC Press, Boca Raton, Fla, USA, 1996.

[9] K. T. Smith and F. Keinert, "Mathematical foundations of computed tomography," *Applied Optics*, vol. 24, no. 23, pp. 3950–3957, 1985.

[10] A. Katsevich, S. Basu, and J. Hsieh, "Exact filtered backprojection reconstruction for dynamic pitch helical cone beam computed tomography," *Physics in Medicine and Biology*, vol. 49, no. 14, pp. 3089–3103, 2004.

[11] J. D. Pack and F. Noo, "Cone-beam reconstruction using 1D filtering along the projection of *M*-lines," *Inverse Problems*, vol. 21, no. 3, pp. 1105–1120, 2005.

[12] J. D. Pack, F. Noo, and R. Clackdoyle, "Cone-beam reconstruction using the backprojection of locally filtered projections," *IEEE Transactions on Medical Imaging*, vol. 24, no. 1, pp. 70–85, 2005.

[13] Y. Ye, S. Zhao, H. Yu, and G. Wang, "Exact reconstruction for cone-beam scanning along nonstandard spirals and other curves," in *Developments in X-Ray Tomography IV*, vol. 5535 of *Proceedings of SPIE*, pp. 293–300, Denver, Colo, USA, August 2004.

[14] Y. Ye and G. Wang, "Filtered backprojection formula for exact image reconstruction from cone-beam data along a general scanning curve," *Medical Physics*, vol. 32, no. 1, pp. 42–48, 2005.

[15] Y. Ye, S. Zhao, H. Yu, and G. Wang, "A general exact reconstruction for cone-beam CT via backprojection-filtration," *IEEE Transactions on Medical Imaging*, vol. 24, no. 9, pp. 1190–1198, 2005.

[16] H. Yu, Y. Ye, S. Zhao, and G. Wang, "A backprojection-filtration algorithm for nonstandard spiral cone-beam CT with an n-PI-window," *Physics in Medicine and Biology*, vol. 50, no. 9, pp. 2099–2111, 2005.

[17] H. Yu, S. Zhao, Y. Ye, and G. Wang, "Exact BPF and FBP algorithms for nonstandard saddle curves," *Medical Physics*, vol. 32, no. 11, pp. 3305–3312, 2005.

[18] S. Zhao, H. Yu, and G. Wang, "A unified framework for exact cone-beam reconstruction formulas," *Medical Physics*, vol. 32, no. 6, pp. 1712–1721, 2005.

[19] T. Zhuang, S. Leng, B. E. Nett, and G.-H. Chen, "Fan-beam and cone-beam image reconstruction via filtering the backprojection image of differentiated projection data," *Physics in Medicine and Biology*, vol. 49, no. 24, pp. 5489–5503, 2004.

[20] Y. Zou, X. Pan, D. Xia, and G. Wang, "PI-line-based image reconstruction in helical cone-beam computed tomography with a variable pitch," *Medical Physics*, vol. 32, no. 8, pp. 2639–2648, 2005.

[21] H. Yu, S. Zhao, and G. Wang, "A differentiable Shepp-Logan phantom and its applications in exact cone-beam CT," *Physics in Medicine and Biology*, vol. 50, no. 23, pp. 5583–5595, 2005.

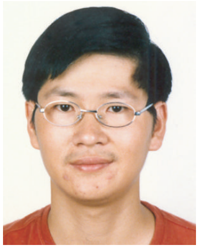

**Hengyong Yu** received his B.S. degree in information science and technology and Ph.D. degree in information and communication engineering from Xi'an Jiaotong University, Xi'an, Shaanxi, China, in July 1998 and June 2003, respectively. He was Instructor and Associate Professor with College of Communication Engineering, Hangzhou Dianzi University, Hangzhou, Zhejiang, China, from July 2003 to September 2004. Currently, he is a Research Fellow with the CT/Micro-CT Laboratory, Department of Radiology, University of Iowa, Iowa City, Iowa, USA. His interests include computed tomography and medical image processing. He authored or coauthored 22 journal papers and 11 conference proceedings. He serves as Guest Editor of the International Journal of Biomedical Imaging for the special issue entitled "Development of Computed Tomography Algorithms." He is a Member of the IEEE and the Chinese Institute of Electronics. He was honored for an outstanding doctoral dissertation by Xi'an Jiaotong University, and received the first prize for a best natural science paper from the Association of Science and Technology of Zhejiang Province.

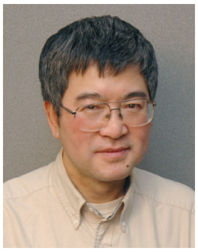

**Ge Wang** received the B.E. degree in electrical engineering from Xidian University, Xi'an, China, in 1982, M.S. degree in remote sensing from Graduate School of Academia Sinica, Beijing, China, in 1985, and M.S. and Ph.D. in electrical and computer engineering from State University of New York, Buffalo, in 1991 and 1992. He was Instructor and Assistant Professor in the Department of Electrical Engineering, Graduate School of Academia Sinica from 1984–1988, Instructor and Assistant Professor in the Mallinckrodt Institute of Radiology, Washington University, St. Louis, Mo, from 1992–1996. He was Associate Professor at the University of Iowa from 1997-2002. Currently, he is Professor in the Departments of Radiology, Biomedical Engineering, Mathematics, Civil Engineering, Electrical and Computer Engineering, and Director of the Center for X-Ray and Optical Tomography, University of Iowa. His interests include computed tomography, bioluminescence tomography, and systems biomedicine. He has published over 300 journal articles and conference papers, including the first paper on spiral/helical cone-beam CT and the first paper on bioluminescence tomography. He is the Editor-in-Chief for the International Journal of Biomedical Imaging, and Associate Editor for IEEE Trans. Medical Imaging and Medical Physics. He is an IEEE Fellow and an AIMBE Fellow. He is also recognized by a number of awards for academic achievements.

